# Student nurses’ experiences regarding their clinical learning opportunities in a public academic hospital in Gauteng province, South Africa

**DOI:** 10.4102/hsag.v25i0.1217

**Published:** 2020-02-17

**Authors:** Mpho N. Motsaanaka, Agnes Makhene, Hafisa Ally

**Affiliations:** 1 Faculty of Health Sciences, University of Johannesburg, Johannesburg, South Africa

**Keywords:** Clinical learning, learning opportunities, experiences, public academic hospital, student nurses

## Abstract

**Background:**

During the training of student nurses, clinical placement is a compulsory requirement, as it exposes them to learning opportunities for the acquisition of clinical skills. This prepares them to become safe and competent professional nurses. However, the increased intake of student nurses in the Gauteng nursing colleges led to overcrowding in a public academic hospital, thus negatively influencing their learning experiences and availability of clinical learning opportunities.

**Aim:**

The purpose was to explore and describe the student nurses’ experiences regarding their clinical learning opportunities to make recommendations to enhance their clinical learning opportunities in order to address the optimisation of their learning experiences.

**Methodology:**

A qualitative, exploratory, descriptive and contextual research design was used. A purposive sampling method was used to select second-year student nurses registered in the Regulation (R425) programme for qualifying as a nurse (general, psychiatry and community) and midwife, as they would have acquired at least 1 year of clinical experience. Four focus groups, which comprised six to eight participants, were constituted, and research was conducted until data were saturated. Field notes were simultaneously taken to enrich the data collected. Thematic coding of qualitative data was used. Principles of trustworthiness and ethical principles were adhered to.

**Results:**

The study revealed four themes. Three were negative experiences that included overcrowding, negative emotional experiences of student nurses and challenges of professional nurses. A theme concerning positive experience entailed knowledge-sharing amongst various health care disciplines.

**Conclusion:**

It was evident that student nurses had more negative emotional experiences than positive experiences. Therefore, the need to enhance their clinical learning opportunities in order to address the optimisation of learning experiences is eminent.

## Introduction

During the training of student nurses, clinical placement in hospitals is a compulsory and an essential requirement, as it exposes students to clinical learning opportunities for acquisition and achievement of clinical skills and competencies. Exposure to clinical practice in hospitals and the development of clinical skills prepare student nurses to become safe and independent professional nurses on graduation. The South African Nursing Council (2005, SANC), *Nursing Act* (No. 33 of 2005), stipulates that student nurses must acquire practical and clinical skills competencies prior to registration as professional nurses. Therefore, it is imperative to expose student nurses to clinical learning opportunities prior to their graduation.

However, the increased intake of student nurses in nursing colleges has led to a large number of students being placed in public academic hospitals. Statistics from Gauteng’s Department of Health and Social Development (2015) have shown a large intake of student nurses in one of the nursing education institutions in Gauteng. From 2010 to 2015, the intake of student nurses increased from 292 to 409 per year, leading to a large number of student nurses being placed in a public academic hospital, consequently affecting the availability of clinical learning opportunities. A clinical learning environment should be rich in learning opportunities to enable student nurses to progress from being a novice to a professional nurse. Clinical exposure to appropriate learning opportunities also develop clinical competency and higher-order thinking skills in student nurses, and assist them with clinical preparedness, as they undergo role transition into the nursing profession (Coyne & Needham 2012).

### Problem statement

Student nurses brought incomplete clinical workbooks after being clinically placed for a specified period. They opined that they did not have sufficient exposure to clinical learning opportunities because of the placement of a large number of students at a public academic hospital, which caters to a variety of students for clinical training. Besides nursing students registered for a diploma programme under Regulation 425 – a multidisciplinary team comprising doctor students, physiotherapy students and other nursing students from private colleges were placed for clinical learning opportunities. This led to overcrowding, creating challenges for student nurses to meet their clinical objectives and poor integration of theory into practice. Subsequently, inadequate clinical learning opportunities led to the extension of training of student nurses. Student nurses are thus not immersed in the nursing profession, and according to SANC, *Nursing Act* (Act No. 33 of 2005), clinical hours for student nurses should not be less than 60% of the entire duration of the course. No empirical contextual evidence could be found as to how overcrowding affected the learning opportunities of student nurses.

The above problem statement led to the following research questions:

- What are the experiences of student nurses regarding their clinical learning opportunities in a public academic hospital?
- What should be carried out to enhance their clinical learning opportunities to address the optimisation of their learning experiences?

### Purpose of the study

The purpose of this study was to explore and describe the experiences of student nurses regarding their clinical learning opportunities in a public academic hospital to further enhance their clinical learning opportunities to address the optimisation of their learning experiences.

### Research design and methods

This study followed a constructivistic paradigm. A qualitative, exploratory, descriptive and contextual research design was used. The participants’ experiences regarding their clinical learning opportunities were explored and described in a systematic, interactive, subjective and holistic approach to give them a meaning (Burns & Grove 2011). This included the description of the population and the sample, data collection, data analysis and measures to ensure trustworthiness. The conceptualisation process involved the exploration of literature to arrive at a meaningful interpretation and concluding statements, which formed the basis for recommendations.

### Population and sample

The population for the study comprised all student nurses registered under the Regulation 425 programme and placed in a public academic hospital. A purposive sampling method was used to select second-year student nurses registered for a diploma programme under Regulation 425 (Gerrish & Lathlean 2015). Thus, information regarding the phenomenon under study could be studied in a way that represents the population of interest. It included the student nurses who met the inclusion criteria and were willing to participate in the study. Inclusion criteria comprised both male and female second-year student nurses placed in a public academic hospital for their clinical training, irrespective of nationality, race, age and culture.

### Data collection

Data collection is a precise and systematic gathering of information relevant to the research purpose and the objectives of the study (Grove, Burns & Gray 2013). Data were collected via focus group interviews by the researcher after the successful completion of research methodology and interviewing skills. Moreover, the researcher was involved in the clinical training of participants. Reflective notes that documented the researcher’s personal values, experience and progress were kept throughout to avoid bias in data collection.

A focus group is a data collection method where groups are assembled to obtain the participants’ perceptions in focussed areas in a setting that is permissive and non-threatening (Krueger & Casey 2015). Twenty-seven participants that took part in this research were divided into four focus groups, each group comprising seven, eight, six and six participants, respectively.

Focus group interviews were audio-recorded with the participants’ permission to assist the researcher to obtain verbatim transcriptions, and diverse and comprehensive data. Field notes were taken to enrich the collected data. Focus group interviews were conducted after structured clinical guidance (SCG) in clinical department in order not to interfere with the students’ learning and clinical training (Krueger & Casey 2015). The duration of the focus group interviews was between 30 and 45 min.

### Data analysis

Thematic coding of qualitative data was used (Holloway & Wheeler 2010). Quotes with similar meaning were grouped together to form themes. An independent coder with knowledge and expertise in qualitative data analysis was used. Thereafter, a consensus meeting between the researcher and the same independent coder was held to verify the accuracy of the data analysed.

## Measures to ensure trustworthiness

Trustworthiness is the degree of being accurate about the data collected using the following.

Lincoln and Guba’s (1985) strategies: credibility, transferability, confirmability and dependability.

Credibility was ensured through prolonged engagement with participants, persistent observation, triangulation, member checking and peer debriefing. Ample time was spent with participants during information sessions to build trust. Information sessions were held prior to focus group interviews for introduction; explaining the research purpose, objectives and significance of the study; and to request voluntary consent to participate in this study. Focus group interviews were conducted until saturation of data and an in-depth understanding of the phenomenon was obtained. The technique of member checking was also used through sharing the findings with the participants to check whether the findings resonated with the participants. A detailed description of the results was provided and supported by direct quotations from the participants. Transferability was facilitated through the provision of a detailed description of the findings to enable prospective researchers to reach a conclusion about whether transfer to other settings could be possible.

Dependability was ensured through a dense description of research methodology, careful documentation during focus group interviews, member checking and the use of an independent coder. Focus group interviews, field notes and audiotape recordings were used as sources of data collection that were explored and used to support emerging themes and subthemes. Stepwise replication method for comparing researcher and independent coder’s findings was used for accuracy. Furthermore, the code–recode strategy was also used where the researcher coded the data, and after a week recoded the same data and evaluated the results (Anney 2014). Confirmability was ensured by outlining the decisions made throughout the research process, and how the data were collected, recorded and analysed. All documents, audiotape recordings and field notes are to be kept for 3 years for cross-checking.

## Ethical considerations

As stipulated in the Republic of South Africa’s (2003) *National Health Act* (Act No. 61 of 2003), Ethics Committee approval from the chief executive officer (CEO) of the preselected hospital and from the university and the higher degree committee was obtained. The researcher created a rapport with the participants to establish a trusting relationship during information sessions. The ethical principles of autonomy and respect for persons were ensured through an information letter, containing the objectives, purpose and benefits of the study, given to the participants prior to their signing of the voluntary informed consent. Additional permission to audio-record focus group interviews was sought and granted by the participants. No harm was inflicted on the participants during the focus group interviews. The researcher also employed the principle of justice in subject selection. Moreover, the participants were informed that they would not be discriminated against should they wish to withdraw and no costs would be charged from them (Dhai & McQuoid-Mason 2011).

## Conceptualisation process

Table 1 gives a summary of the findings. Four themes and related subthemes emerged from the findings.

**TABLE 1:** Themes and related subthemes.

Themes	Subthemes
1. Experiences of overcrowding	1.1. Difficulties in managing a large number of students by professional nurses 1.2. Competing for clinical procedures by the students of various disciplines
2. Negative emotional experiences	2.1. Anger and frustration 2.2. Lack of motivation
3. Challenges of professional nurses	3.1. Lack of support and supervision by professional nurses 3.2. Poor interpersonal relationships between college lecturers, student nurses and professional nurses
4. Positive experiences of knowledge-sharing with and from various health care disciplines.	

### Theme 1: Experiences of overcrowding by students

Participants reported negative experiences of overcrowding. They observed that professional nurses had difficulty in managing large number of students from various disciplines, and students were competing for clinical procedures. Their negative experiences of overcrowding in clinical areas resulted in decreased clinical learning opportunities. A participant articulated:

‘Mam, one other thing is that currently in midwifery, we are more than the patients. So, we end up crowding the patient. We even find it difficult to assess the patient.’ (Participant 3 in focus group 4 verbatim)

Killam and Heerschap (2013) posited that an overcrowded clinical facility because of a large number of students is not a conducive environment for learning, as it hinders students’ effective clinical learning and opportunities to practice their skills. This overcrowding by students of various health care disciplines, such as medical, pharmacy, physiotherapy and nursing in a clinical setting, impedes their clinical training and learning opportunities, resulting in a lack in achieving the clinical objectives (Nabolsi et al. 2012).

#### Subtheme 1.1: Difficulty in managing large number of students

Participants reported that professional nurses appeared to find it difficult to manage a large number of students. This negatively influenced their clinical learning because professional nurses could not give individual attention to students, as they were required to perform their daily duties to provide quality nursing care. Participants also reported that professional nurses in wards allocated them previous year’s clinical outcomes and non-nursing tasks. This further limited their clinical learning opportunities. The following verbatim of a participant reflected this issue:

‘It becomes difficult for the professional nurses to manage us; they end up not knowing what to do with us because we are so many in the ward.’ (Participant 5 in focus group 1 verbatim)

The above is in line with Jacobs, Vakalisa and Gawe (2012) who reported that a large number of students in clinical areas could create difficulty in managing and maintaining good discipline, giving individual attention and opportunities to practice. Furthermore, failure to provide such learning opportunities results in a nursing graduate with non-competency in clinical skills and required nursing standards (O’Mara et al. 2014).

#### Subtheme 1.2: Competing for clinical procedures by students from various disciplines

The public academic hospital selected for this study caters to students from various disciplines, namely, student doctors, physiotherapists and nurses from both government and private academic institutions. Participants expressed having a negative experience because of competing clinical learning opportunities. The participant articulated:

‘It’s like we are competing for the skills. How do you do abdominal palpation on one patient when it is us and student doctors?’ (Participant 3 in focus group 4)

Shuid et al. (2015) stated that the presence of many students in a clinical setting from various disciplines lead to overcrowding that decreased clinical training and learning opportunities for student nurses.

### Theme 2: Student nurses had negative emotional experiences

Gagné (1965) was quoted from Hughes and Quinn (2013) reporting that emotions and attitude of the learner as important factors to ensure positive and successful learning. Botma et al. (2014) argued that negative emotional experiences can limit and block students’ opportunities to pursue learning. They describe negative emotions as personality variables that include stress, anxiety, depression, frustration and contempt. Participants expressed negative emotions of anger, frustration and lack of motivation that emanated from a lack of clinical learning opportunities. A participant reported as follows:

‘I never got an opportunity to do anything. One of the professional nurses even said to me, “I am a nuisance.” She does not have time for me.’ (Participant 1 in focus group 4)

Rasepae (2013) similarly found that a threatening learning environment influences one’s emotions negatively, delaying the ability to seek opportunities, focus and pay attention to learning.

Participants further reported that wards were understaffed and professional nurses were overworked, which led to students not being adequately exposed to clinical learning and opportunities to practice skills.

Negative emotions of stress and poor staff morale in professional nurses because of being overworked, caused lack of interest in students’ teaching, thus further limiting students’ clinical learning opportunities. A participant verbalised as follows:

‘They actually don’t have the interest to teach us. She was just sending me to give the patients medication without allowing me next to the medication trolley.’ (Participant 2 in focus group 3)

The findings are in line with the study conducted by Williams (2017), which indicated that the health care environment was at a great risk of incivility because of stressful working conditions.

**FIGURE 1:**
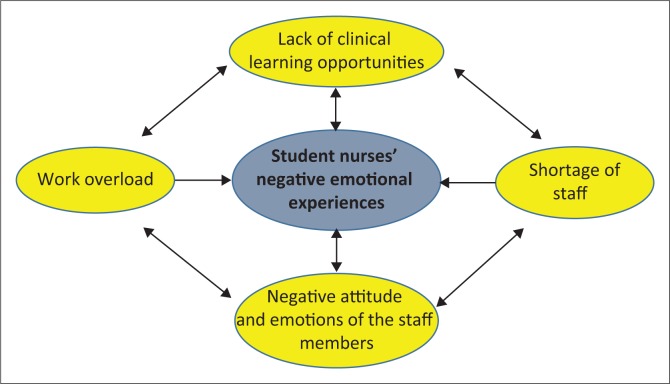
Cyclic relationship between the negative emotional experiences of the students and the staff.

The student nurses’ lack of clinical learning opportunities resulted in negative emotional experiences, exacerbated by a negative attitude and emotions of professional nurses in wards. The negative attitude and emotions of professional nurses was because of work overload caused by shortage of staff in wards. The noted cyclic relationship between student nurses and the staff’s attitude and experience is summarised in Figure 1.

#### Subtheme 2.1: Anger and frustration

Botma et al. (2014) defined anger as a strong negative emotional response and a feeling of displeasure that often represents ethical dilemma. Frustration is the result of blocked motivation drive that prevents one from reaching the desired goal (Botma et al. 2014). Participants reported that professional nurses showed less interest in their process of teaching and were not eager to impart knowledge. As a result, students expressed emotions of anger and frustration because they were deprived of learning opportunities. A participant articulated:

‘They want you to feel that you are a student. They even make comments like: we’ll see, she won’t even get to D4:4.’ (Participant 6 in focus group 3)

(D4:4 means a fourth [final]-year nursing student registered in Regulation [R425] programme leading to become a nurse [general, psychiatry and community] and midwife). Buthelezi et al. (2015) concur that lack of interest from the professional nurses nurses limited the opportunities for the student nurses to practice nursing skills and becoming clinically competent.

Participants further expressed their anger and frustration as professional nurses used them to push workload in wards, further resulting in missed learning opportunities. The students felt that they were not treated as students and were used as extra pair of hands. The participant said:

‘When we are in the wards, we are pushing work and not learning anything.’ (Participant number 3 in focus group 3)

Another participant added:

‘I was so frustrated in that ward mam. She didn’t allow me to do the dressing. I was so angry because she just used me and was also telling me to get other stuff for her as if I’m her secretary.’ (Participant 1 in focus group 3)

Bisholt et al. (2014) agreed that student nurses were used as extra pair of hands, leading to minimal exposure to learning experience, thus causing frustration amongst nursing students.

#### Subtheme 2.2: Lack of motivation

Motivation is the desire to achieve a task optimally and is indicative of a person’s positive disposition (Burton & Ormrod 2011). Nabolsi et al. (2012) concurred that motivation is the cornerstone in fulfilling one’s learning and intended outcomes. Participants expressed their lack of motivation using notions such as discouraged and not interested, because of professional nurses’ negative attitude towards them, low staff morale and lack of learning opportunities. The latter resulted in students becoming less and less motivated. A participant reported:

‘The professional nurses’ attitude towards us is so demotivating mam. They believe we are only there in clinical areas to stress them and *re ba pitlaganya le bophelo* (we are causing traffic).’ (Participant 4 in focus group 4)

Another participant added:

‘We didn’t have so many patients. I went to town and came back without the professional nurses noting that I was not there.’ (Participant 2 in focus group 2)

Similarly, Rikhotso, Williams and De Wet (2014) reported that there was a high turnover and absenteeism of student nurses who were unmotivated and discouraged, leading to decreased clinical exposure to learning opportunities.

On the contrary, some students sought learning opportunities to ensure that their clinical learning objectives are met. As the participant commented. They reported that despite overcrowding in clinical areas, they sought opportunities to ensure that their clinical objectives are met. As one participant commented:

‘It is also my responsibility to learn and seek opportunities in the wards to ensure that I learn something. When other students saw the opportunity to dodge, I used it positively and asked the professional nurses to send me to another ward in order to expose myself to other learning opportunities.’ (Participant 4 in focus group 2)

These sentiments resonated with Jack’s (2017) findings which concurred that student nurses ended up with the need to be resilient and developed strategies to seek learning opportunities despite the challenges they encountered in clinical areas.

### Theme 3: Challenges of professional nurses

Participants articulated the challenges that professional nurses faced at workplaces that negatively affected student nurses’ clinical learning opportunities as lack of support and supervision, poor interpersonal relationships between college lecturers, professional nurses and student nurses.

#### Subtheme 3.1: Lack of support and supervision by professional nurses

Botma et al. (2014) described support as creating effective learning opportunities and availing all necessary resources to maximise students’ learning experiences. Support in clinical areas should be provided by training institutions, workplace and lecturers to benefit all parties involved. Participants expressed lack of support and supervision from professional nurses in wards as a negative experience that reduced their learning opportunities in clinical areas. The participant articulated:

‘They (professional nurses) don’t have time to teach us and they don’t take teaching as their responsibility. The first thing they like asking us is ‘didn’t they show you at college’? Basically, we are the college responsibility and not theirs.’ (Participant’s 3 verbatim in focus group 1)

Participants further reported that understaffed wards and overworked professional nurses, aggravated by the presence of a large number of students, led to professional nurses becoming unsupportive, with no time to teach, provide learning opportunities and supervise student nurses.

A participant articulated:

‘Professional nurses in the wards are short-staffed and overworked. When we ask for their supervision to practice skills, they are too tired or too busy to help us.’ (Participant 2 in focus group 1)

The heavy workload and the negative attitude of the professional nurses towards students affected negatively the students’ learning opportunities (Rajeswaran 2016). Killam and Heerschap (2013) added that this resulted in student nurses carrying out procedures alone without any supervision from professional nurses, and resulted in loss of learning and growth opportunities.

#### Subtheme 3.2: Poor interpersonal relationships between college lecturers, student nurses and professional nurses

Poor interpersonal relationships can lead to disharmony and unhappiness amongst group members, which can cause lack of achievement of the set goals (Jooste 2010). Participants reported poor interpersonal relationships and lack of communication between college lecturers, student nurses and professional nurses as contributing factors that created negative clinical experiences, blocking opportunities to learning and achieving their objectives.

A participant articulated:

‘In this other ward, the staff members’ attitude towards each other was so bad. And I was caught in the middle because I didn’t know who to report to without offending the other.’ (Participant 6 in focus group 2)

Even Rajeswaran (2016) reported poor interpersonal relationships and lack of effective communication between nurse educators and clinical/professional nurses in clinical areas. This atmosphere frustrated student nurses as they were not appropriately placed in clinical areas to ensure adequate exposure to learning opportunities in order to meet their clinical learning outcomes.

### Theme 4: Positive experiences of knowledge-sharing with and from various healthcare disciplines

Hughes and Quinn (2013) defined knowledge-sharing as bringing together individuals from different disciplines, with the aim of providing opportunities to learn with and from one another. It is also sharing of knowledge, clinical training resources and team-ship amongst different professionals. The Nursing and Midwifery Council (2015, NMC), London, postulated that the programme providers must ensure that students have the opportunity to learn with and from other health and social professionals in practice and academic settings.

Participants reported knowledge-sharing with and from various healthcare disciplines as a positive experience, as it exposed them to varied clinical learning opportunities.

They reported how other healthcare disciplines assisted them in their clinical learning and opportunities.

One participant verbalised:

‘One time, the doctor called me to try and put up a drip. Even though I was scared, he was there with me. The fact that he let me practice was enough for me.’ (Participant 3, verbatim focus group 3)

This strong support and positive attitude towards inter-professional learning promotes knowledge-sharing amongst students who may broaden the need for interdisciplinary clinical learning (Echarri-Martinez et al. 2011). Interdisciplinary learning encompasses learning from the following healthcare disciplines involved in the care of patients: nursing, medicine, physiotherapy and nutrition dietetics (Laskowski-Jones 2016).

## Recommendations

Table 2 outlines the recommendations based on the findings of the study, as nursing students had more negative emotional experiences regarding their clinical learning opportunities. Recommendations are with reference to nursing education, practice and further research.

**TABLE 2:** Recommendations after/post data analysis.

Recommendations	Data analysis
1. Nursing education	Formulate communication policies and guidelines between the college and the hospital with regard to student nurses’ clinical placement in order to enhance clinical learning and objectives. Assessment and accreditation of other potential healthcare institutions and the optimal use of all wards in academic hospitals for equal distribution, diverse learning opportunities and positive experience. Identify strategies to assist students to be actively involved in their own learning, develop learning and coping strategies, for example, referral to the students’ counselling body. Benchmarking and development of a programme with other healthcare disciplines that will fit the academic hospital to enhance and support interdisciplinary learning.
2. Nursing practice	Recruit, appoint and improve retention strategies (e.g. encourage professional development and the use of positive rewards) to address the shortage of professional nurses with appropriate skill-mix in hospitals. Promote a student-friendly environment to transform negative emotional experiences of student nurses into positive emotional experiences. Avail all resources to assist in student nurses’ learning, for example, improving staff distribution to reduce staff workload and related stress. Engage all stakeholders in continuing professional development in order to maintain and enhance professional standards, competency and best practices. Professional nurses should be incentivised for teaching, supervision and provision of learning to student nurses. This will boost staff morale to be actively involved in student nurses’ learning. Allocation of on-site clinical lecturers and mentors to support and supervise students. Promote in-service training amongst operational managers on different management styles that will assist in creating a positive learning environment. Revisit and re-implement the ‘grand rounds’ (multidisciplinary team rounds) in hospitals to appreciate and promote knowledge-sharing.
3. Nursing research	Further research is recommended in other public and private academic hospitals in Gauteng to evaluate if the same results could be replicated. Further research to be undertaken to explore and evaluate if recommendations are viable. Conduct a study on nursing management views of clinical learning experiences of student nurses.

## Limitations of the study

The study was contextual in nature; therefore, the findings could not be generalised. Focus group interviews were conducted in afternoons after SCGs. Participants might have been too tired to reflect critically on their clinical experiences from the previous year to the time of data collection. Some participants were not as active as the others. They acknowledged that they were afraid that professional nurses would treat them badly if they (student nurses) reported about the professional nurses to the college lecturers. Participants were reassured of confidentiality and anonymity during data collection and analysis. Some participants used it as an opportunity to raise their general concerns and the challenges they face in the hospital.

## Conclusion

The study revealed that student nurses had more negative than positive experiences. The negative experiences included overcrowding by students, student nurses’ negative emotional experiences, challenges of working with professional nurses, and positive experiences of knowledge-sharing with and from various healthcare disciplines. Recommendations were made to promote positive learning experiences that might enhance student nurses’ clinical learning opportunities in a public academic hospital, as nursing is always considered a practice-based profession. The nursing practice might benefit if clinical learning opportunities are enhanced and learning experiences of student nurses are optimised. Student nurses need to have adequate exposure to clinical learning opportunities in order to produce independent, professional nurses with higher-order thinking skills.

## References

[CIT0001] Anney V.N., 2014, ‘Ensuring the quality of the findings of qualitative research: Looking at trustworthiness criteria’, *Scholarlink Research Institute Journals* 5(2), 272–281.

[CIT0002] Bisholt B., Ohlsson U., Engström A.K., Johansson A.S. & Gustafsson M., 2014, ‘Nursing students’ assessment of the learning environment in different clinical settings’, *Nurse Education in Practice* 14(3), 304–310. 10.1016/j.nepr.2013.11.005 24355802

[CIT0003] Botma Y., Brysiewics P., Chipps J., Mthembu S. & Phillips M., 2014, *Creating stimulating learning opportunities*, 1st edn., Pearson Education South Africa, Cape Town.

[CIT0004] Burns N. & Grove S.K., 2011, *Understanding nursing research. Building evidence-based practice*, 5th edn., Elsevier Saunders, Philadelphia.

[CIT0005] Burton R. & Ormrod G., 2011, *Nursing: Transition to professional practice*, 1st edn., Oxford University Press, New York.

[CIT0006] Buthelezi S.F., Fakude L.P., Martin P.D. & Daniels F.M., 2015, ‘Clinical learning experiences of male nursing students in a bachelor of nursing programme: Strategies to overcome challenges’, *Curationis* 38(2), 1–7. 10.4102/curationis.v38i2.1517 PMC6092699 26842075

[CIT0007] Coyne E. & Needham J., 2012, ‘Undergraduate nursing students’ placement in specialty clinical areas: Understanding the concerns of the student and registered nurse’, *Contemporary Nurse* 42(1), 97–104. 10.5172/conu.2012.42.1.97 23050576

[CIT0008] Department of Health and Social Development, 2015, *Statistics, 2015*, DoH, Gauteng.

[CIT0009] Dhai A. & McQuoid-Mason D., 2011, *Bioethics, human rights and health law. Principles and practice*, 1st edn., Juta, Cape Town.

[CIT0010] Echarri-Martinez L., Fernandez-Llamazares C.M., Manrique-Rodriguez S., Garcia-Lopez I., Lopez-Herce J. & Sanjurjo-Saez M., 2011, ‘Pharmaceutical care in pediatric intensive care unit: Activities and interdisciplinary learning in a Spanish hospital’, *European Journal of Hospital Pharmacy* 19, 416–422. 10.1136/ejhpharm-2011-000032

[CIT0011] Gerrish K. & Lathlean J., 2015, *The research process in nursing*, 7th edn., Wiley Blackwell Malaysia, Kuala Lumpur.

[CIT0012] Grove S.K., Burns N. & Gray J.R., 2013, The *practice of nursing research. Appraisal, synthesis and generation of evidence*, 7th edn., Elsevier, Philadelphia.

[CIT0013] Holloway I. & Wheeler S.W., 2010, *Qualitative research in nursing and healthcare*, 3rd edn., Wiley Blackwell, London.

[CIT0014] Hughes S.J. & Quinn F.M., 2013, *Quinn’s principles and practice of nurse education*, 6th edn., Cengage Learning, Andover.

[CIT0015] Jack K., 2017, ‘The meaning of compassion fatigue to student nurses: An interpretive phenomenological study’, *Journal of Compassionate Health Care* 4(2), 1–8.

[CIT0016] Jacobs M., Vakalisa N.C.G. & Gawe N., 2012, *Teaching-learning dynamics*, 4th edn., Pearson Education, Cape Town.

[CIT0017] Jooste K., 2010, *The principles and practice of nursing and health care. Ethos and professional practice, management, and research*, 1st edn., Van Schailk, Pretoria.

[CIT0018] Killam L.A. & Heerschap C., 2013, ‘Challenges to student learning in the clinical setting: A qualitative descriptive study’, *Nurse Education Today* 33(6), 684–691. 10.1016/j.nedt.2012.10.008 23141689

[CIT0019] Krueger R.A. & Casey M.A., 2015, *Focus groups. A practical guide for applied research*, 5th edn., Sage, Thousand Oaks, CA.

[CIT0020] Laskowski-Jones L., 2016, ‘Interdisciplinary education: Learning together from the same playbook’, *Wolters Kluwer* 46(4), 1–6. 10.1097/01.NURSE.0000481428.41189.06 26945148

[CIT0021] Lincoln S.Y. & Guba E.G., 1985, *Naturalistic inquiry*, Sage, Beverley Hills, CA.

[CIT0022] Nabolsi M., Zumot A., Wardam L. & Abu-Moghli F., 2012, ‘The experience of Jordanian nursing students in their clinical practice’, *Procedia-Social and Behavioral Sciences* 4, 5849–5857. 10.1016/j.sbspro.2012.06.527

[CIT0023] Nursing and Midwifery Council (NMC), 2015, *Standards for competence for registered nurses*, NMC, London.

[CIT0024] O’Mara L., McDonald J., Gillespie M., Brown H. & Miles L., 2014, ‘Challenging clinical learning environments: Experiences of undergraduate nursing students’, *Nurse Education in Practice* 14(2), 208–213. 10.1016/j.nepr.2013.08.012 24063792

[CIT0025] Rajeswaran L., 2016, ‘Clinical experiences of nursing students at a selected institute of health sciences in Botswana’, *Health Science Journal* 10(6), 1–6. 10.21767/1791-809X.1000471

[CIT0026] Rasepae K.M.M., 2013, ‘Facilitating learning through humour at a nursing education institution in Gauteng’, *University of Johannesburg* 1–102.

[CIT0027] Republic of South Africa, 2003, *The National Health Act, Act No.: 61 of 2003*, Government Printers, Pretoria.

[CIT0028] Rikhotso S.R., Williams M.J.S. & De Wet G., 2014, ‘Student nurses’ perceptions of guidance and support in rural hospitals’, *Curationis* 37(1), 1–6. 10.4102/curationis.v37i1.1164 26852424

[CIT0029] Shuid A.N., Yaman M.N., Kadir R.A.A., Hussain R.I., Othman S.N., Nawi A.M. et al., 2015, ‘Effects of early clinical skills teaching on third year medical students’ learning: The student perspective’, *Journal of Taibah University Medical Schemes* 10(1), 26–32. 10.1016/j.jtumed.2014.12.004

[CIT0030] South African Nursing Council, 2005, *Nursing Act No.: 33 of 2005*, Government Printers, Pretoria.

[CIT0031] Williams T.L., 2017, ‘Student incivility and its impact on nursing faculty and the nursing profession’, *Walden Dissertations and Doctoral Studies* 1–150.

